# Understanding the Non-Surgical Treatment Experience of Female Patients with Carpal Tunnel Syndrome: A Qualitative Study

**DOI:** 10.3390/ijerph191912349

**Published:** 2022-09-28

**Authors:** Paloma Moro-López-Menchero, Cristina García-Bravo, César Fernández-de-las-Peñas, Javier Güeita-Rodríguez, Carmen Jiménez-Antona, Domingo Palacios-Ceña

**Affiliations:** 1 Department of Physical Therapy, Occupational Therapy, Rehabilitation and Physical Medicine and Research Group of Humanities and Qualitative Research in Health Science (Hum&QRinHS), Universidad Rey Juan Carlos, 28922 Madrid, Spain; 2 Department of Physical Therapy, Occupational Therapy, Rehabilitation and Physical Medicine and Research Group of Manual Therapy, Dry Needling and Therapeutic Exercise (GITM-URJC), Universidad Rey Juan Carlos, 28922 Madrid, Spain

**Keywords:** carpal tunnel syndrome, doctor–patient relationship, women, qualitative research

## Abstract

Carpal tunnel syndrome (CTS) is a peripheral neuropathy of the upper extremity, characterized by pain, loss of strength, and decreased fine motor function. This study describes the experiences of women with CTS who received non-surgical treatments. A qualitative phenomenological study was undertaken. Purposive sampling was used. Women with clinical and electromyographic diagnoses of CTS were included. Eighteen in-depth interviews were conducted among women with CTS, and field notes were kept. The Giorgi’s approach was used for qualitative analysis of the data collected. Five themes emerged: (a) Seeking help and waiting for a diagnosis, (b) trying non-surgical therapeutic options, (c) avoiding invasive options, (d) treatment expectations, and (e) relationships with clinicians. The women described how diagnoses were delayed because women delay seeking help and referrals to medical specialists. Women avoid surgical options and prefer to opt for more conservative approaches, such as splinting or physical therapy. The main reason for avoiding surgical treatment is the fear of limitations and that surgery will not fully eliminate the symptoms. Conflicts may arise in the relationship with the clinician, and they demand to be able to participate in the decision-making process.

## 1. Introduction

Carpal tunnel syndrome (CTS) is a peripheral neuropathy of the upper extremity, representing 90% of all neuropathies [1]. Spain has an incidence of 4.2 cases per 100,000 workers, affecting 62.8% of women [2]. An estimated 4–5% of the world’s population suffers from CTS, with an incidence of 6% in men and 9.2% in women [3]. Risk factors for systemic CTS include diabetes, pregnancy, obesity, hypothyroidism, renal and heart failure, and autoimmune diseases [3,4]. Other mechanical factors include activities that force the use of the wrist in flexion–extension positions, overuse of wrist flexor muscles, post-traumatic deformities, or being subjected to prolonged hand vibrations [3,4].

The clinical presentation of CTS is characterized by pain, paresthesia in the areas of nerve innervation with worsening at night, atrophy of the thenar eminence muscles (in severe cases), loss of strength, and decreased fine motor coordination [1]. Women are more likely to suffer from CTS, even in absence of associated comorbidities, presenting greater thenar muscle atrophy and edema, and better postoperative outcomes compared to men [5].

Three stages of evolution are described, depending on the severity of the symptoms. [3]. In the first initial stage, patients suffer nocturnal symptoms that awaken them with numbness and tingling of the hands and fingers, and/or pain extending from the wrist to the shoulder (brachialgia paresthetica nocturna) [1,3]. In the second phase, symptoms worsen, occur diurnally, and appear when maintaining certain hand positions for a long time, or when performing repetitive activities with the hands, such as drawing, writing, and screwing. In addition, in this phase, the patients begin to have difficulties grasping objects, causing them to drop objects [3]. In the third, advanced stage, symptoms are constant and there is atrophy of the thenar muscles and/or weakness in thumb abduction, and fine motor coordination decreases [1,3].

Based on the intensity and/or severity of symptoms, treatment can be conservative (exercises and manual therapy) or surgical. Surgical treatment has high healthcare costs, influencing the degree of satisfaction with the care received [6]. Fernández-de-las-Peñas et al. described the results of a clinical trial comparing the effects of surgery against manual physical therapy (including desensitization techniques of the central nervous system combined with tendon/nerve gliding exercises) in women with CTS [7]. This clinical trial found that both treatment approaches exhibited similar results at a follow-up period up to 4-years [7]. In a similar direction, Shi et al. [8] reported that patients with CTS treated with surgery showed only small improvements compared to those receiving physiotherapy; however, surgery was associated with greater side effects and complications than more conservative treatments.

Qualitative studies conducted in patients with CTS have described (a) their experiences in relation to their improvements and side effects following treatments received [9], (b) their return to work after surgery [10], and (c) their expectations about the surgical treatments received [11,12,13]. Recently, Moro-López-Menchero et al. [14] explored the experiences of women with CTS regarding the management of symptoms and limitations. However, at present, the qualitative literature on CTS is still scarce. Aspects, such as the meaning of the disease, acceptance and choice of treatment, decision making, and the relationship with healthcare professionals during the treatment, need to be studied. At present, no qualitative study has described the experiences of women with CTS in relation to non-surgical treatments, and the motivations for selecting these treatments. The objective of this study was to describe the experience of women with CTS who received non-surgical treatments regarding their diagnoses and treatment options, their expectations, reasons for accepting or refusing them, and their relationships with their clinicians.

## 2. Methods

### 2.1. Design

A qualitative phenomenological study [15,16] based on Husserl’s framework [15,17] was conducted. Qualitative studies are used in order to achieve a deeper understanding of people’s behaviors under certain specific circumstances, such as rehabilitation and pain disorders [16]. Qualitative studies may also be used, to know the perspective of patients and their families regarding the effects of health interventions [17,18]. The data obtained via qualitative research comes from data collection tools, such as interviews, focus groups, and participant observation, and in the form of narrative transcriptions, images (drawings, photography), and documents (diaries, letters) [19]. The field of qualitative studies involves phenomenology attempts to understand people’s lived experiences by using first-person narratives and other sources, such as personal letters and diaries [17].

### 2.2. Research Team and Reflexivity

Six researchers (50% women) participated in this study, including one research nurse (DPC), and five physical therapists. All researchers had experience in research in health sciences and none had any care relationship with the patients at the time of the study. The investigators had prolonged engagement with the study setting and participants during data collection and analysis.

### 2.3. Participants, Setting, and Sampling Strategies

Patients with CTS attending the Neurology Departments of Hospital Universitario Fundación Alcorcón were recruited between November 2019 and February 2021. Participants were identified and recruited from the neurology service consultation. The patients chose not to have surgery and came to a physical therapy practice seeking clinical support and alternative treatments. In phenomenological studies, it is common to include participants based on purposive sampling [18,19]. Purposive sampling can be defined as the selection of individuals based on specific purposes associated with addressing the research study question or aim [16,18,19]. Participants who met the inclusion criteria were recruited consecutively from the neurology consultation, and data collection ceased when the information gained from the interviews became repetitive [18,19]; in our study this situation occurred after including 18 participants.

The study included females aged 18–65 years old, who did not receive previous surgical treatments and were diagnosed with CTS according to clinical and electromyographic criteria. The clinical criteria included paresthesia and/or pain in the territory innervated by the median nerve, positive Tinel’s test, and Phalen’s test [3]. Electro-diagnostic criteria will consider motor and sensory conduction deficits of the median nerve according to criteria established by the American Society of Electromyography, American Academy of Neurology, and American Academy of Physical Medicine and Rehabilitation. The exclusion criteria were: (a) previous treatment with surgery and/or steroids; (b) trauma to the upper limb; (c) diagnosis of diabetes mellitus or other diseases that explained the symptoms; and (d) musculoskeletal disease (rheumatoid arthritis, sympathetic-reflex dystrophy, fibromyalgia).

### 2.4. Data Collection and Management

Based on the phenomenological design, first person data collection tools (unstructured and semi-structured interviews) and researcher field notes were used simultaneously [18,19]. The first phase of data collection was performed via unstructured interviews based on the following opening question: what is your experience with CTS? During the second phase, a question guide was elaborated using the data obtained in the unstructured interviews that took place in the first phase (participants 1 to 9), and which were used as the bases for the format of the semi-structured interviews (Table 1). In the second phase, semi-structured interviews were used (participants 10–18) to obtain information regarding specific issues of interest.

During the interviews, researchers used prompts for clarifications (paraphrasing something that the participant had said) and to encourage the participant to provide further details (‘Can you tell me a bit more about that?’). This enabled relevant information to be collected from the participants’ perspectives.

All of the interviews (*n* = 18) were tape-recorded and transcribed verbatim, recording a total of 484 min of interviews. Additionally, 18 field notes were collected by the researchers during interviews since field notes provide a rich source of information, i.e., participants describe their personal experiences, their behaviors during data collection, and enable their reflections to be noted concerning methodological aspects of the data collection [19]. The interviews were held in a private room at the university by PMLM, DPC, and CJA, and no third party was present. There were no dropouts.

### 2.5. Analysis

The thematic framework method using the model proposed by Amadeo Giorgi [17,20] was used for data analysis. This model distinguishes five stages of data processing: (1) data collection; (2) reading, prior literal transcription of the interviews; (3) breaking down the descriptions into separate units in order to identify the relevant meaning units for the phenomenon under study; (4) data organization and listing from the perspective of the discipline using a process of encoding; and (5) data synthesis and summarization to communicate these to the scientific community. Double and independent coding were performed by two investigators (PMLM, DPC). The independent coding consisted of two investigators performing coding separately and without sharing coding files. Both had experience in qualitative studies in health sciences.

The data obtained were analyzed separately for both the unstructured and semi-structured interviews. Subsequently, in both phases, a coding grid was created with the meaning units, their groups, and the identified themes. Within this grid, the narrations that justified the results obtained were identified [21]. Thereafter, group sessions were performed among the two researchers. In these sessions the themes obtained in both phases were analyzed and compared. Subsequently, the final themes were obtained and were decided via researcher consensus. No qualitative software was used to analyze the data. For the analysis, the Excel program was used to organize and share the coding process.

### 2.6. Rigor

The guidelines for conducting qualitative studies established by the Consolidated Criteria for Reporting Qualitative Research [22] and the Standards for Reporting Qualitative Research [23] were followed. The techniques performed and application procedures used to control trustworthiness [24] are described in Table 2.

### 2.7. Ethics

The study was approved by the Local Ethical Committee of Universidad Rey Juan Carlos (code: 0806202014020). All participants provided informed consent prior to their inclusion.

## 3. Results

Eighteen women with CTS were enrolled in this study with a mean age of 40 years (SD 10). The participants presented a symptom evolution over time of 23.3 months (SD 11.6), all had nocturnal symptoms, 44.44% (*n* = 8) had symptoms when driving, and a mean pain intensity (measured from 0 to 10) at the time of the interview of 4.8 (SD 1.5). The patients had a mean time of diagnosis of CTS of 15.1 months (SD 10.8). Table 3 details demographic and clinical features of the sample.

Five main themes were identified: (a) seeking help and waiting for the diagnosis, (b) trying non-surgical therapeutic options, (c) avoiding invasive options, (d) treatment expectations, and (e) relationship with clinicians. Table 4 shows a summary of the coding tree, with meaning units identified from narratives with relevant information, groups of common meanings or groups of meaningful units (similar points or content that allowed the emergence of the topics that described the study participants’ experiences), and finally the main themes.

In order to facilitate the traceability and identification of the results obtained, below, each theme is accompanied by excerpts of transcripts. These narratives enrich and justify the qualitative results [23].

### 3.1. Seeking Help and Waiting for the Diagnosis

Our participants related how the first professional they turned to for diagnosis was the primary care physician. This professional is the filter for the referral to a medical specialist in the Spanish public health system. Most of the participants were referred to traumatologists and neurologists, and on rare occasions, rheumatologists.

The reasons for seeking help included repetitive symptoms, increased pain intensity and duration over time, trauma to the hand, and inability to adapt the workstation:

“… then I decided to go to the doctor, because I couldn’t stand it, and it was getting harder and harder for me to do things.”.

(P10)

Our participants described how the delay in the diagnosis was determined, partly, by their delay in seeking professional help, and also by the delayed referral to the medical specialist and the performance of diagnostic tests by the health system:

“…first, I had to wait for the primary care doctor send me to the neurologist, about three months, then it was the appointment of the specialist to do the EMG test, about six to eight months, and then when they did everything, almost a year for the diagnosis since I asked for help.”.

(P15)

Regarding diagnosis and therapeutic options (need for surgery), the information was perceived by the participants as unclear and somewhat confusing:

“The diagnosis hasn’t been clear; I don’t know if I need surgery. The traumatologist told me that he isn’t ready to operate, that I should take vitamins and wear a splint and in three months he will assess it... what exactly?”.

(P3)

Once the diagnosis was made, the only treatment offered to participants with severe cases was surgery, and in less severe cases, conservative treatments were prescribed, such as night splints, medication, stretching, and physiotherapy. Invasive treatments such as infiltrations were also proposed. In the case of surgery, although it was the most frequent option proposed by the specialist physicians, it was also the one most frequently rejected by our participants:

“…I don’t want to have surgery; they can’t assure you that everything will function properly after the surgery…”.

(P4)

### 3.2. Trying Non-Surgical Therapeutic Options

Our participants described the different non-surgical proposals of CTS. Our participants recounted how they considered these measures to be conservative only because they were non-surgical. One of the most commonly used treatment approaches was night splints. Thus, the splint is usually prescribed by both types of clinicians (specialist and primary care physicians), although sometimes participants purchased the splint based on recommendation by the pharmacist’s third party or on their own account:

“When the doctor recommended me to use the splint, I had already been using it for a few weeks. I was advised by my neighbor who has the same one, and by the neighborhood pharmacist.”.

(P17)

The participants reported that although it was indicated for use at night, they wore the splint during the day. The criterion for daytime use was the presence of pain. Most of the participants reported that, when they first put the splint on, it bothered them, and was uncomfortable; however, over time they felt relief, symptoms decreased, and the presence of nighttime symptoms, such as cramps, pain, and numbness, improved, allowing them to rest. Our participants described how, despite the fact that the splints were recommended by the doctor, their applications were delayed due to several reasons. The financial situation and the low incomes of the participants led them to prioritize other daily needs rather than buying the splint:

“… The doctor told me it was good for me to get [the splints] so I could sleep. But with my pay roll, I don’t have enough to cover my daily needs, so I haven’t been able to buy them.”.

(P12)

On other occasions, the use of splints was delayed because the women preferred to try other treatment alternatives, such as physiotherapy. Instead of the splint, a soft wrist bandage, a bandage, or Kinesio taping, were used in the presence of pain or when the women wanted to make their problems visible at work or within the family. Our participants reported that being perceived as patients was important to them and influenced their acceptance of different treatments:

“… there have been days when I didn’t need to wear the wristband and I have worn I all the same, so that people are aware that I can’t do all the things I did before…”.

(P1)

Physiotherapy was the most common private option because it was the most accessible option in their neighborhoods and surroundings. On a few occasions, the physician prescribed physiotherapy services in the public health system. In these cases, a limited range of techniques were applied, causing participants to feel that the full potential of physical therapy was not being used to solve their problems. Physiotherapy treatments included strengthening exercises, stretching exercises, electric currents, and manual therapy.

Among the pharmacological options, they were prescribed analgesics, anti-inflammatory drugs, and, on a few occasions, vitamins. The majority reported little or no improvement with medication. Many of our participants complained about the lack of specific and alternative medical treatments, when their severity was not great enough to undergo surgery. They felt as if they were up against the wall, torn between either accepting surgical treatment or accepting medical treatment that did not resolve their symptoms:

“…the options are very limited, surgery if you are sick, and if you are not very sick, to put up with it and wait for it to get worse. He didn’t tell me anything specific.”.

(P4)

### 3.3. Avoiding Invasive Options

The reasons given by our participants for limiting options that were considered invasive, such as infiltrations, surgery, and the use of drugs, are described. These reasons were based on their personal needs, fears, and previous negative experiences. In addition, the participants claimed that the opinions and information from their social environments (family, neighbors) influenced their decisions.

Our participants declined and avoided invasive measures, such as infiltrations and surgery. The reason they avoided infiltrations was because of pain. They refused to accept them in order to eliminate their CTS pain; they had to undergo more pain during treatment administration (infiltrations):

“… If the pain becomes more acute, they will do infiltrations that will give me more pain. Obviously, I want to avoid them.”.

(P17)

On other occasions, they followed the recommendations of third parties in their environments who advised them to avoid infiltrations because they were not “good”. In these cases, the participants acknowledged that trust in these people (e.g., other professionals) was very important to them:

“…I go to a private physio to avoid another infiltration because I was told they were not good... I do not know very well why they are not good for, but I did not infiltrate again.”.

(P1)

All participants categorically rejected surgery. They explained that they did not want to undergo surgery and sought to delay it for as long as possible. Fear was an important factor; fear of going into the operating room, fear of losing autonomy and depending on their family and friends, fear of losing their jobs, and fear of having to be re-intervened at some point down the road. They also reported other fears regarding post-surgical recovery times, or being away from family and having no one to turn to or ask for help:

“…I am afraid. My daughters depend on me a lot, because of my job. When I have the operation, I won’t be able to rest to recover, I will have to go back to work.”.

(P14)

Moreover, surgery was perceived as a risk in itself—a risk that should be left as a last therapeutic resource. The participants described how they preferred to exhaust all possible therapeutic options before surgery. Some women pointed out that the lack of information about surgery, the reasons for its application, possible consequences, and prognoses made them feel distrustful about this option.

Finally, in CTS, the consumption of medications was perceived as unwarranted because they had no clear effect, and they did not have curative effects on the disease:

“… the pills cure one thing and spoil four things, so it’ s best to avoid them, […] they give it to you because of course it will give you some relief, but it’ s not advisable.”.

(P6)

### 3.4. Treatment Expectations

For our participants, a good treatment outcome was associated with a reduction of pain, or pain disappearing, avoiding surgery, improving the functionality of their hands in activities, such as writing, picking up objects, performing their work normally, resting, being able to sleep, and adopting body positions (doing physical activity) where they could put weight in their hands without pain:

“…to have a good quality working life, because I work a lot with my hands, having strength in my hands is important to me, because I have to use them a lot.”.

(P8)

“…To sleep through the night, that’s my greatest luxury in life which was taken away from me because of the carpal tunnel problem.”.

(P16)

Moreover, a good outcome meant not being dependent on anyone when it came to performing their daily living and work activities and becoming completely independent again. Our participants reported that maintaining their autonomy in relation to child and family care was a key expectation for them.

Conversely, a poor outcome was associated with pain, symptoms remaining or returning after a temporary improvement, medical services forgetting about them, not being able to apply any other therapeutic options, or having to undergo surgery:

“…the worst thing would be for the pain to increase, for it to increase and for them to forget about me.”.

(P9)

“…when they offer you surgery, they don’t guarantee that they are going to take away the pain. That’s the bad thing, that they can’t give you any further treatment, and you continue to have pain for the rest of your life.”.

(P18)

Most of the participants pointed out the difficulty of finding a single professional who was able to offer therapeutic options that would meet all of their expectations.

### 3.5. The Relationship with Clinicians

Most of the participants pointed out that when they asked for more information from primary care doctors and specialists regarding the possible origin of CTS, measures to prevent its worsening, and/or progression, they were not provided with any concrete information because the injury could no longer be prevented.

Sometimes, conflicts with doctors arose when participants demanded medical reports to be presented to the company’s occupational risk services, to request a work adaptation due to the CTS or sick leave:

“… he wouldn’t even give me a written piece of paper with what happened to me. And I need it because I must take it to the company. If I leave because I can’t stand the pain anymore, the company must know that it’ s been painful for a long time.”.

(P1)

Our participants highlighted that they had no role in the decision making or in the possibility of being offered more therapeutic options. The doctors had already made the decisions beforehand. The decision was always made without any option to change:

“…The doctor hasn’t even suggested that I do exercises or go to a physiotherapist. Nobody gives you options, well yes, one, the usual one, surgery, and they have already decided for you.”.

(P2)

Our participants narrated that factors that affected their relationships with their clinician included failure to listen to the patient’s needs/questions, limiting the choice of treatment to only two options (accepting or refusing surgery), not giving other non-surgical treatment possibilities, lack of time during the specialist’s consultation, and not giving the patient time or the option to ask questions:

“…The doctor who examined me did not listen to me. She just wanted me to tell her if I was going to have surgery or not. When I told her no, she told me that I was therefore opting out of the operation, that I should inform the primary care medical doctor and that if the clinical situation worsened, I should come back”.

(P1)

## 4. Discussion

Our results show that participants first seek help from the primary care medical doctor, and this is where the referral to the specialist is made. Schwartz et al. [25] described how the primary care doctor is the first health professional to start with conservative therapies and coordinating with other specialists for more invasive treatment proposals.

In relation to other treatments, such as the use of drugs, Arcury et al. [26] noted that the most commonly used medication for the relief of CTS symptoms was ibuprofen and vitamins. Our participants did not feel improvement in their pain or other symptoms with drugs and avoided continued pharmacological treatment.

Our results show how the participants rejected the application of infiltration. Nonetheless, infiltration is a useful alternative when conservative treatments fail, or when the surgical option is not desired [25]. Additionally, corticosteroid injections decrease wrist swelling and vascular congestion [9]. Platelet-rich plasma injections are now beginning to be used [25,26].

We found that the use of a splint comes from a medical prescription or without a prescription, on the advice of a third party. Immobilization with prefabricated rigid splints, generally used at night, is the most frequent initial conservative treatment prescribed to patients with CTS [27,28]. The splint immobilizes and reduces wrist swelling, improving symptoms [28]. In fact, Atroshi et al. [27] described how when starting to wear the splint, patients may notice discomfort and limitation, but subsequently feel relief and decrease in pain, and tingling. Atroshi et al. [27] also showed that patients may present difficulties investing money in these devices.

Our participants rejected surgery because of fear of different factors, such as incomplete functional recovery, remission of symptoms, or loss of autonomy. Mansilla et al. [11] describe how after undergoing surgery for CTS, patients report being satisfied with the improvements and increased physical dexterity. Klokkari and Mamais [9] showed how surgical treatment improves symptoms, hand functionality, and neurophysiological parameters, as compared to conservative treatment. Moreover, both treatments are effective for improving symptoms and functionality at six months, although conservative treatment has fewer complications than surgery.

Regarding the doctor–patient relationship, Belasen et al. [29] reported that good doctor–patient communication is essential for the patient to trust the doctor. In addition, these authors [29] point out that in patients with CTS it generates better adherence to treatments, better coping with the disease, presenting fewer complications, and requiring up to 50% fewer diagnostic tests. However, our participants reported that the doctor did not listen to them, did not give them time to ask their questions, or did not even offer them a report to take back to the company. Harbishettar et al. [30] noted that a patient’s distrust of the clinician results in frequent physician switching, losing the therapeutic benefit of the clinician–patient relationship.

Our participants narrated that they were not offered recommendations, or other options, such as physical therapy. Gong et al. [31] described how the decision-making process in treatment choice between patients with CTS and their medical doctors should be based on patient preference and their expectations about treatment outcomes. These authors [31] recommend a joint physician–patient decision-making model, provided the patient is previously correctly informed of risks and benefits of each intervention, and the treatment options are within each patient’s personal interests.

Finally, the authors of the current study believed that the female experience regarding CTS is relevant because situations of gender inequality may exist regarding the distribution of roles in the home, childcare, and household chores and affect women’s health. Jonsson et al. [32] describe the phenomenon of a “double burden” in women, which consists of having a paid job outside the home, and in addition, they continue to carry the burden of home care and childcare. This may cause some women with CTS to reject surgery as a treatment because they are responsible for the care of the home and family [14].

The strength of the present study is that it presents results on a topic that has been scarcely studied to date. Among the limitations, the results cannot be extrapolated due to the nature of the design. Secondly, the present study has included 18 participants, and 18 interviews have been performed. Previous qualitative studies [15,19,33] describe how the total number of participants included does not depend on a previous calculation of the sample size, rather it is based on the saturation or redundancy of the information obtained in the interviews. Turner-Bowker et al. [34] reported that 92–97% of the saturation can be analyzed after interview numbers 15 and 20. In addition, this study only focused on women and therefore, future research should extend it to men and compare their experiences. At present, there is little or no research using qualitative methodology, from the exclusive perspective of men and/or women with carpal tunnel syndrome. Moro-López-Menchero et al. [14] and Mansilla et al. [11] exclusively included women in their studies. Whereas other studies included mixed samples of men and women, such as Newington et al. [10], Arcury et al. [26], Khu et al. [13], and Jerosch-Herold et al. [12].

## 5. Conclusions

Our results show how a group of women with CTS experienced the process of diagnosis, the choice of non-surgical treatment, and their relationships with healthcare clinicians. These results can help rehabilitation professionals to understand the needs of women with CTS when prescribing different types of treatments and establishing therapeutic relationships.

## Figures and Tables

**Table 1:** Semi-structured interview guide.

Issue/Topic	Questions
Disease	How would you describe your pain and your condition? What do you know about the disease you suffer from? What aspects of the disease are most relevant to you? What does the disease mean to you?
Diagnosis	What prompted you to seek medical help? Can you explain the process of your diagnosis? What is your opinion regarding the time it took to reach a diagnosis for your symptoms?
Treatment	What treatment(s) have you received and has it solved your problem? What do you consider to be the most relevant aspect of the treatment that has been prescribed to you? What are your thoughts regarding the treatment? Do you adhere to the treatment? Why? What do you expect from the treatment(s)? What expectations of a cure do you have?

**Table 2:** Trustworthiness criteria.

Criteria	Techniques Performed and Application Procedures
Credibility	Investigator triangulation: each interview was analyzed by two researchers. Team meetings were performed in which the analyses were compared, and categories and themes were identified. Triangulation of methods of data collection: unstructured, semi-structured interviews were conducted, and researcher field notes were kept. Member checking: asking the participants to confirm the data obtained at the stages of data collection. All participants were offered the opportunity to review the audio and/or video records to confirm their experience. None of the participants made additional comments.
Transferability	In-depth descriptions of the study were performed, providing details of the characteristics of researchers, participants, contexts, sampling strategies, and the data collection and analysis procedures.
Dependability	Audit by an external researcher: an external researcher assessed the research protocol, focusing on aspects concerning the methods applied and study design. An external researcher specifically checked the description of the coding tree, the major themes, participants’ quotations, identification of quotations, and descriptions of themes.
	Investigator triangulation, member checking, and data collection triangulation.
Confirmability	Researcher reflexibility was encouraged via the performance of reflexive reports and by describing the rationale behind the study.

**Table 3:** Demographic and clinical features.

	Age	Sex	Diagnostic Time (Months)	Duration of Symptoms (Months)	Affected Side	Pain Intensity	Type of Work	Treatments before Recruitment
P1	47	Female	48	48	Right	7	Clothing Clerk	Splint, wristband, kinescoping, and physiotherapy
P2	36	Female	12	18	Bilateral	5	Chef	Rigid wristband and stretching
P3	43	Female	6	7	Left	6	Housewife	-
P4	24	Female	12	24	Bilateral	5	Shop Manager	-
P5	35	Female	24	24	Right	5	Occasional worker	Splint and injection
P6	40	Female	7	36	Right	6	Housekeeper	-
P7	32	Female	12	24	Right	5	Waitress	Splint and exercise
P8	50	Female	18	24	Bilateral	3	Midwife	Splint
P9	22	Female	5	12	Bilateral	5	Clothing Clerk	Splint and exercise
P10	54	Female	6	6	Bilateral	8	Civil Servant	Splint, paraffin bath and exercise
P11	45	Female	18	24	Bilateral	3	Clothing Clerk	-
P12	43	Female	5	5	Bilateral	6	Housekeeper	Splint, paracetamol, and injection
P13	32	Female	18	18	Right	3	Optician	Splint, physiotherapy and exercise
P14	29	Female	12	36	Left	5	Clothing Clerk	Bandage
P15	47	Female	24	30	Bilateral	6	Civil Service Administrator	Splint, physiotherapy, anti-inflammatory, and exercise
P16	56	Female	24	30	Bilateral	3	Computer Expert	Splint
P17	49	Female	3	18	Bilateral	3	Massage Therapist	Splint and anti-inflammatory
P18	37	Female	18	36	Bilateral	3	Hairdresser	Splint, physiotherapy, and medication

**Table 4:** Summary of the coding tree.

Meaning Units	Groups of Common Meaning	Themes
Professional performing the diagnosis Delay of diagnosis Delay factors (symptoms, increased pain intensity and duration) Characteristics of diagnostic information	Diagnosis	Seeking help and waiting for the diagnosis
Triggers for seeking help Professionals they turn to	Seeking help	
First therapeutic options offered Severe vs. mild cases Surgery and other invasive measures Other non-invasive measures	Therapeutic options	
Medical prescription of the splint Daytime splint use Discomfort versus symptom improvement and rest Delayed application	Night splint	Trying non-surgical therapeutic options
Splint replacement Disease visibility	Soft wrist support or bandage	
Most commonly used non-pharmacological treatment Techniques and forms of treatment	Physiotherapy	
Use of analgesics and/or anti-inflammatory drugs	Drugs	
Rejection due to pain in its administration Rejection due to advice from third parties	Infiltrations	Avoiding invasive options
Refusal of surgery Reasons for refusal (fear, loss of autonomy) Uncertainty about post-surgical recovery Perception of risk Last therapeutic resort	Surgery	
Too little effect Too much of risk in relation to benefit	Medication consumption	
Avoidance of surgery Improved hand functionality Rest and sleep Being independent and maintaining autonomy	Good treatment outcome	Treatment expectations
Physician abandonment Ending up in surgery	Poor treatment outcome	
Limited information on alternatives to surgical treatment Gaps in information	Medical information	Relationship with clinicians
Absence of medical reports for sick leave No opinion in decision making No therapeutic options	Conflict with physicians	
Lack of willingness to listen Limited choice of treatments No possibility of non-surgical treatment Lack of time in consultation No option to ask questions	Poor relationship with the physician	
